# 703 Extubation Success in Pediatric Burn Patients: A 10-Year Review

**DOI:** 10.1093/jbcr/irad045.179

**Published:** 2023-05-15

**Authors:** Richard Cartie, Balakrishna Prasad, Noah Pierzchajlo, Austin Price

**Affiliations:** Burn and Reconstructive Centers of America, Augusta, Georgia; Eisenhower Army Medical Center, Fort Gordon, Georgia; Mercer University of Medicine, Savannah, Georgia; Joseph M. Still Research Foundation, Augusta, Georgia; Burn and Reconstructive Centers of America, Augusta, Georgia; Eisenhower Army Medical Center, Fort Gordon, Georgia; Mercer University of Medicine, Savannah, Georgia; Joseph M. Still Research Foundation, Augusta, Georgia; Burn and Reconstructive Centers of America, Augusta, Georgia; Eisenhower Army Medical Center, Fort Gordon, Georgia; Mercer University of Medicine, Savannah, Georgia; Joseph M. Still Research Foundation, Augusta, Georgia; Burn and Reconstructive Centers of America, Augusta, Georgia; Eisenhower Army Medical Center, Fort Gordon, Georgia; Mercer University of Medicine, Savannah, Georgia; Joseph M. Still Research Foundation, Augusta, Georgia

## Abstract

**Introduction:**

Pediatric burn care presents two unique population subsets that require distinctive care from adult and non-burn patients, respectively. While prior research has led to the creation of best practices for burned adults and non-burn pediatric patients, the standard of care regarding long-term pediatric burn intubation varies widely and deviates from accepted standards in the non-burn pediatric world. In this retrospective review, we analyze one burn center’s experience managing ventilation and extubation of pediatric patients.

**Methods:**

We reviewed all pediatric patients admitted to the burn and wound care ICU who were intubated for more than 24 hours. Patient data was collected from 2012 through 2022. 442 patients were initially identified and 142 remained after removing screen failures. We analyzed 3 groups which included total burn surface area (TBSA), inhalation injury, and time on the ventilator.

**Results:**

The first group broken down by TBSA yielded the most significant results in our 7 of 10 metrics. All significant metrics including mortality, length of stay (LOS), and pulmonary hypertension (HTN) were directly correlated to burn TBSA. Patients with higher TBSA were also significantly more likely to require reintubation after coming off the ventilator. Inhalation injury presented on admission was also a significant indicator for developing acute respiratory distress syndrome (ARDS). Patients who remained on the ventilator for >7 days were significantly more likely to develop pulmonary HTN, however mortality and reintubation rates notably did not increase in this group.

**Conclusions:**

There is much debate in the burn world as to whether standard practices should carry over from other specialties when treating severely burned patients. Much of our data confirms, in pediatric patients, findings of adult patients found in the literature. Mortality, LOS, and time on the ventilator expectedly increased with burn TBSA. Inhalation injury was a significant indicator for ARDS which implies that proper screening for this type of injury is a key to predicting long-term treatment. What is perhaps most notable in this data is the fact that, unlike in the adult burn population, there was no significant difference in mortality between short and long-term intubations. Additionally, patients who required reintubation (n=7) were significantly more likely to have a TBSA >40%, but those who remained on the ventilator >7 days were not more likely to require intubation after extubation. This implies that patients should be left on the ventilator, if necessary, without resorting to aggressive challenge breathing trials for early extubation as is common in adult population. With this data we will also identify our rare cases of pediatric tracheostomy placement and indications for its use.

**Applicability of Research to Practice:**

This projects shows how intubation of pediatric burn patients can be maintained safely and effectively in the critical care setting.

